# Leaf litter quality drives the feeding by invertebrate shredders in tropical streams

**DOI:** 10.1002/ece3.6169

**Published:** 2020-07-27

**Authors:** Guilherme Sena, José Francisco Gonçalves Júnior, Renato Tavares Martins, Neusa Hamada, Renan de Souza Rezende

**Affiliations:** ^1^ AquaRiparia/Lab. de Limnologia Department of Ecology University of Brasilia Brasilia Brazil; ^2^ Graduate Program in Ecology University of Brasilia Brasilia Brazil; ^3^ Coordenação de Biodiversidade Instituto Nacional de Pesquisas da Amazônia—INPA Manaus Brazil; ^4^ Program of Postgraduate in Ecology and Evolution Universidade Federal de Goiás—UFG Goiânia Brazil; ^5^ Program of Postgraduate in Environmental Sciences Community University of Chapecó Region Chapecó Brazil

**Keywords:** Amazonia, aquatic insect, Cerrado, detritus quality, quantity of organic matter, riparian zone

## Abstract

Amazon and Cerrado‐forested streams show natural fluctuations in leaf litter quantity along the time and space, suggesting a change on litter quality input. These natural fluctuations of leaf litter have repercussion on the organic matter cycling and consequently effects on leaf decomposition in forested streams. The effects of the quantity of leaf litter with contrasting traits on consumption by larvae of shredder insects from biomes with different organic matter dynamics have still been an understudied question. The Trichoptera *Phylloicus* spp. is a typical shredder in tropical headwater streams and keep an important role in leaf litter decomposition. Here, we assessed the consumption by shredder *Phylloicus* spp., from Amazonia and Cerrado biomes, on higher (*Maprounea guianensis*) and lower quality leaves (*Inga laurina*) in different proportions and quantities. Experiments were performed concomitantly in microcosms approaches, simulating Cerrado and Amazonian streams. Higher leaf consumption occurred in Cerrado microcosms. Litter quantity influenced negatively leaf consumption by shredders in Cerrado, in opposition to Amazonia, where consumption was not affected by leaf quantity. In both sites, we observed higher consumption by shredders in treatment with only *M. guianensis* and no difference between other treatments with mixture of leaves. In treatment with litter of *I. laurina,* we noted the use of substrate for case building (due to the higher leaf toughness)*,* affecting the fragmentation process. Therefore, our results indicate that leaf litter quality drives the preference of consumption by *Phylloicus* larvae in Cerrado and Amazonia streams.

## Graphical abstract

Our study performed in two Brazilian biomes showed a higher consumption by shredders in treatment with just the better quality leaf litter and no difference between other treatments with mixture of leaves, demonstrating how leaf litter quality is important for breaking down the organic matter. In addition, our results help to explain how the availability of leaf litter might affect its use by Phylloicus in the Cerrado, in opposition to the from Amazon. Therefore, our results indicate that leaf traits drive the preference of litter consumption by Phylloicus larvae in Cerrado and Amazonia streams.

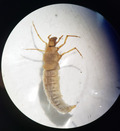

## 1 INTRODUCTION

The availability of resources in ecosystems is one of the factors that determines the spatial distribution of organisms, being able to govern the processes of the ecosystem (Mikola, Bardgett, & Hedlund, 2002). Riparian zones of tropical biomes show natural seasonal changes on litter quantity input (Rezende et al., 2016; Sales, Goncalves, Dahora, & Medeiros, 2015), with a strong seasonality in Cerrado‐forested streams (Tonin et al., 2017). Furthermore, there is a higher input of litter in Amazon than in the Cerrado‐forested streams, related to precipitation as a limiting factor (Tonin et al., 2017). These natural seasonal changes on litter input suggest a change of litter quality input (Gonçalves & Callisto, 2013; Tonin et al., 2017), as well as repercussions for the flow of energy and the cycling of organic matter in streams (Bueno et al., 2016).

Riparian zone closes the canopy above streams (Gonçalves & Callisto, 2013), reducing the light input in lotic ecosystems and, consequently, the primary productivity of the system (Vannote, Minshall, Cummins, Sedell, & Cushing, 1980). Therefore, the main source of energy for low‐order lotic systems comes from the input and subsequent decomposition of litter material originated from the riparian plants, between 60% and 80% (Bambi et al., 2017; Esteves & Gonçalves, 2011) derive from vertical and lateral contribution of the stream edges, respectively (Rezende et al., 2016). Leaf litter in these ecosystems can be retained and accumulated on the riverbed (Bambi et al., 2017), where can undergo leaching of soluble compounds (e.g., tannins and polyphenols; Graça et al., 2015) and be colonized by microorganisms in the conditioning process (Graça, Hyde, & Chauvet, 2016). Finally, invertebrates fragment leaves to use them as food resource and/or shelter (Gessner, Chauvet, & Dobson, 1999; Graça et al., 2015). Detritivore invertebrates (mainly shredders) can dramatically increase leaf breakdown rates (Graça et al., 2015; Moulton, Magalhaes‐Fraga, Brito, & Barbosa, 2010) showing the importance of the shredders for functioning of headwater streams systems (Graça, 2001).

In general, shredders have been found in low abundance in tropical streams (more adapted to lower temperatures; Boyero et al., 2011; Prather, 2003), but due to high biomass and body size they can be important for cycling of organic matter and energy flow in aquatic ecosystems (Martins, Melo, Gonçalves, & Hamada, 2015; Rezende, Petrucio, & Gonçalves, 2014; Tonin, Hepp, Restello, & Gonçalves, 2014). The genus *Phylloicus* Müller, 1880 (Trichoptera: Calamoceratidae) has approximately 60 species, distributed from South to Central America. *Phylloicus*' larvae are commonly found on submerged leaf litter (Prather, 2003). It is the most abundant shredder taxon in Cerrado (Rezende et al., 2014, 2015) and Amazonian headwater streams (Martins, Melo, Gonçalves, & Hamada, 2014; Martins et al., 2015). *Phylloicus* is a typical shredder and uses leaf litter deposited in pool areas on streams bed to obtain their food and material to case construction (Wantzen & Wagner, 2006). In this process, *Phylloicus* larvae convert the coarse particulate organic matter into fine particles and dissolved organic matter (Prather, 2003). This highlights the importance of understanding factors that change the leaf litter processing by *Phylloicus*, and consequently, the functioning of ecosystem.

Ferreira et al. (2015) analyzed the diet of *Phylloicus* larvae and pointed out that the particulate organic matter (fine and coarse) consumed varied among instars with the proportion of food related to stream characteristics. These results indicated the complex diet and the direct connection with local and temporal resource availability (input of leaf litter), highlighting the need for further studies. Events such as temporal changes of litter quantity and the physical environment have been claimed to affect *Phylloicus* populations (Leite, Silva, Navarro, Rezende, & Gonçalves Júnior, 2016; Rezende, Santos, Medeiros, & Gonçalves, 2017). As well, intrinsic leaf traits, such as leaf litter size (Rezende et al., 2018), hardness and nutrients (Biasi, Cogo, Hepp, & Santos, 2019; Graça, 2001), lignin (Gessner, 2005), and phenolic concentrations (Moretti, Loyola, Becker, & Callisto, 2009) are factors that denote the leaf litter quality and explain shredders feed preference. However, biological interactions as competition (Rezende et al., 2015) and risk of predation (Navarro, Rezende, & Gonçalves, 2013) can affect negatively the population dynamics of *Phylloicus.* Leaf litter processing by *Phylloicus* larvae can also be negativity influenced by environmental conditions, as increase of temperature (Martins, Melo, Gonçalves, Campos, & Hamada, 2017; Martins, Rezende, et al., 2017; Navarro et al., 2013). Nevertheless, all these mentioned studies just refer to single populations, using leaf litter and larvae just from the same place in their design.

Previous studies just investigated food preference of *Phylloicus* larvae (Moretti et al., 2009; Navarro et al., 2013). However, the effects of leaf litter quantity of different leaf traits on leaf litter consumption in biomes with different organic matter dynamics have still been an understudied question. Our aim was to assess consumption by *Phylloicus* larvae from different geographic origins on higher (*Maprounea guianensis*) and lower quality leaves (*Inga laurina*) of different proportions and quantities under controlled conditions of microcosms. This experiment will allow assessing of possible impacts on leaf litter processing and survival of *Phylloicus* spp. by changes in Amazonia and Cerrado riparian forest composition. Our hypotheses were: (a) Due to the shredder feeding preference for higher quality leaf litter (Biasi et al., 2019; Rincón & Martínez, 2006), a superior consumption will be recorded in more *M. guianensis* proportion treatments; (b) A higher leaf litter availability will decrease consumption of harder leaf litter, because consumers tends to potentialize the feed in resources that are energetically better for metabolism maintenance (Kaspari, Donoso, Lucas, Zumbusch, & Kay, 2012).

## 2 METHODS

### 2.1 Sampling site description

We sampled *Phylloicus* spp. larvae in Amazonian and Cerrado streams. In Cerrado, larvae were sampled in Capetinga 3rd order stream (15°57′40″S, 47°56′33″W), located in Gama‐Cabeça de Veado watershed of Federal District of Brazil. In Amazonian stream, *Phylloicus* larvae were collected in Barro Branco 2nd order stream, at the Ducke Reserve (02°55′ and 03°01′S, 59°53′ and 59°59′W), Central Amazonia in the Amazon State of Brazil. In the sampling sites, we measured, the stream channel (Cerrado: 2 m; Amazonia: 1.5 m), depth (Cerrado: 0.20 m; Amazonia: 0.1 m), dissolved oxygen (Cerrado: 7.0 ± 0.6 mg/L standard error; Amazonia: 6.6 ± 0.1 mg/L), electrical conductivity (Cerrado: 16.7 ± 0.5 µS/cm^2^; Amazonia: 10.7 ± 0.4 µS/cm^2^), temperature (Cerrado: 19.5 ± 1.0°C; Amazonia: 24.5 ± 0.5°C) and pH (Cerrado: 6.1 ± 0.1; Amazonia: 4.6 ± 0.1).

### 2.2 *Phylloicus*' sampling and identification

**Figure 1:**
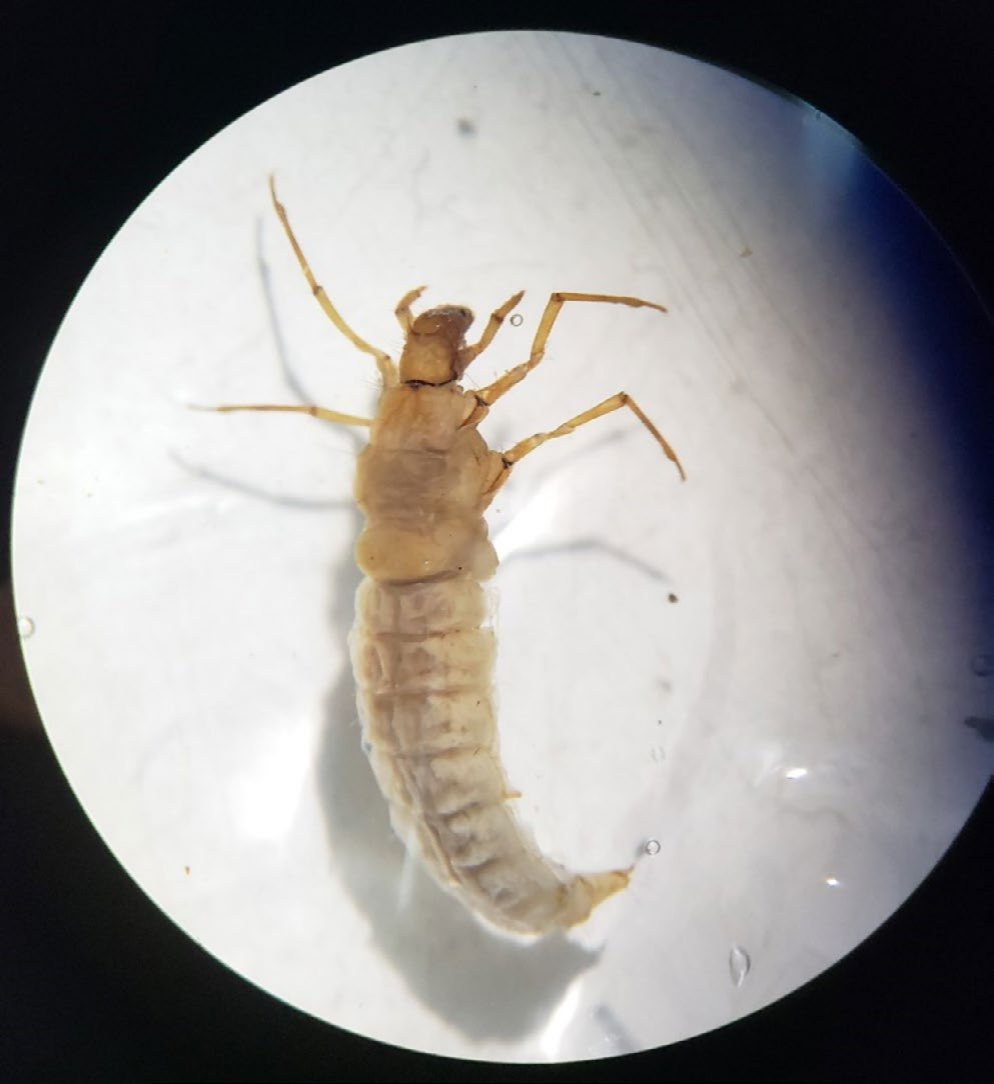
Picture of *Phylloicus* sp. (Trichoptera: Calamoceratidae) observed electronic magnifier glass

*Phylloicus* spp. larvae (Figure 1) were sampled by “active searching” (saw target species and then collected by kicknet) by 4 hr of sampling effort (single day). For identification, some *Phylloicus* larvae from Cerrado stream were collected in the field and kept, individually, in containers with small portion of leaves collected in the field and stream water with constant aeration, each container was covered with fine mesh of 0.5 mm held by a rubber band. Daily observations were made until newly hatched adults were observed. They were captured, preserved in 80% alcohol, and sent to the National Institute for Research in Amazonia (INPA) for identification. The specimens from Cerrado represent an undescribed *Phylloicus* species. Larvae collected in the Amazonian stream belong to the species *Phylloicus elektoros* Prather, association with adult was made by rearing procedures, however, the larva is not formally described yet.

### 2.3 Laboratory procedures

The experiments were performed in the microcosms of the Laboratory of Limnology/AquaRiparia in Department of Ecology at the University of Brasilia (UnB) for the Cerrado biome experiment, and in the Laboratoty of *Citotaxonomia e Inetos Aquáticos* located at the Instituto Nacional de Pesquisas da Amazônia (INPA) for Amazonian biome experiment. *Phylloicus* larvae were taken to the laboratory in cool boxes and placed in containers (15.5 cm × 15.5 cm × 12 cm, 2 L volume) with mineral water and previously calcined gravel (in an oven for 4 hr at 550°C) on the bottom (Martins, Melo, et al., 2017; Martins, Rezende, et al., 2017). Larvae were kept in containers in an experimental room with continuous aeration at a temperature of 20°C and with a light/dark ratio of 12/12 hr throughout the experiment. Daily, we measured dissolved oxygen (Lenway 970 Meter DO2; Cerrado: 6.9 ± 0.3 standard error mg/L; Amazonia: 6.6 ± 0.22 mg/L), electrical conductivity (Lenway 430 pH/cond. Meter; Cerrado: 20.1 ± 1.5 µS/cm^2^; Amazonia: 72.8 ± 5.4 µS/cm^2^) and pH (Lenway 430 pH/cond. Meter; Cerrado: 6.5 ± 0.04; Amazonia: 4.7 ± 0.1) in all containers. *Phylloicus* larvae had their cases removed before the start of the experiments to avoid the consumption of external organic matter (e.g., consumption of case).

Leaf litter of *Maprounea guianensis* and *Inga laurina* were conditioned in litterbags (0.5 mm mesh) for seven days at Capetinga and Barro Branco streams for Cerrado and Amazonia experiments, respectively. As described for Gomes, Medeiros, and Gonçalves (2016)*, Maprounea guianensis* (higher quality leaf litter) presents 23% of lignin, 15% of cellulose, 36% of polyphenols, 0.76% of Nitrogen (N), 0.01% of Phosphorus, and 31 of Lignin:N ratio. *Inga laurina* (lower quality leaf litter) presents 45% lignin, 33% cellulose, 7% polyphenols, 1% N, 0.11% P, and 43 of Lignin:N ratio. Following Gomes et al. (2016), the leaves were classified according to the feed preference of the shredder in relation to leaf hardness, lignin and cellulose concentration and lignin: N ratio in low and high quality for *I. laurina*, and *M. guianensis*, respectively. Both species show large occurrence in riparian vegetation zones throughout the tropical system in Brazil. Subsequently, the conditioned leaves were cut into disks (1.98 cm diameter) and freeze‐dried (Terroni, LT‐ AISI 304 model). Sets of disks were weighed on a precision balance (0.01 mg; Shimadzu, AUW220D model) to determine the initial dry mass and distributed among the treatments. At the end of the experiments, the leaf disks were removed, dried at 60°C for 48 hr and subsequently weighed to obtain the final dry weight. The mass leaf loss (MLL) between treatments was calculated by the difference between the initial and final dry mass, after seven days of experiment in both locals (Figure 1).

### 2.4 Experimental design

**Figure 2:**
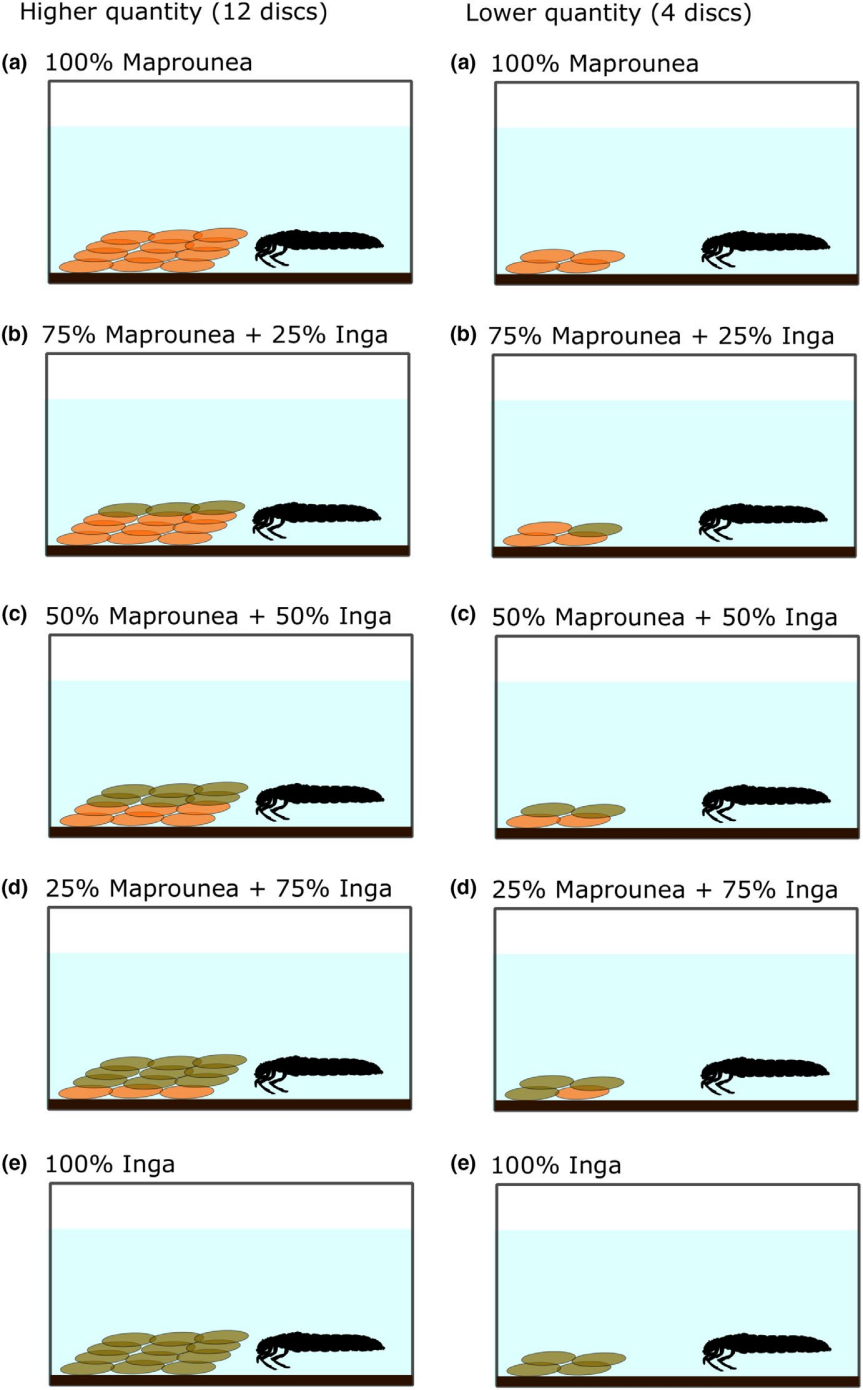
Experimental design performed with 80 containers (Cerrado = 40; Amazonia = 40; four replicates per treatment) to assess the effect of litter quality and quantity on the processing by *Phylloicus* spp. Quality test (a = 100% of *Maprounea guianensis*; b = 75% of *M. guianensis* and 25% of *Inga laurina*; c = 50% of both; d = 25% of *M. guianensis* and 75% of *I. laurina*; and e = 100% of *I. laurina*), in a quality gradient to high (a) to low (e). Quantity test: these five proportions were repeated four for the quantity test, ordered with four leaf disks (Lower quantity) and the second with 12 leaf disks (Higher quantity) in each container

In the experiment, we used 80 containers (Cerrado = 40; Amazonia = 40) with a single *Phylloicus* larvae to avoid interactions between individuals (e.g., aggression and competition) that could interfere in the consumption results. The effect of litter quality was assessed by providing leaf litter of *M. guianensis* and *I. laurina* in five different ratios of number of disks (A = 100% of *M. guianensis*; B = 75% of *M. guianensis,* and 25% of *I. laurina*; C = 50% of both; D = 25% of *M. guianensis* and 75% of *I. laurina*; and E = 100% of *I. laurina*) to *Phylloicus* larvae. These five ratios were repeated in two treatments regarding the quantity of organic matter where the first with four leaf disks (Lower quantity) and the second with 12 leaf disks (Higher quantity) in each container highlight that there were 4 replicates per quantity × quality treatment level in the text (as shown in Figure 2).

### 2.5 Statistical analysis

Through Factorial‐ANOVA between biomes resource availability (leaf quantity), litter proportions (leaf quality) and interaction of these factors (independent variables), we tested the percentage of leaf litter used as food resource by *Phylloicus* (dependent variable). The data were transformed whenever necessary with arcsine square root of the ratio to obtain the best fit (Crawley, 2007). We used contrast analysis to discriminate among statistically significant categorical variables (Rezende et al., 2018). All analyses were conducted in R (R Core Team, 2019).

## 3 RESULTS

### 3.1 Leaf litter quantity and quality effects

**Figure 3:**
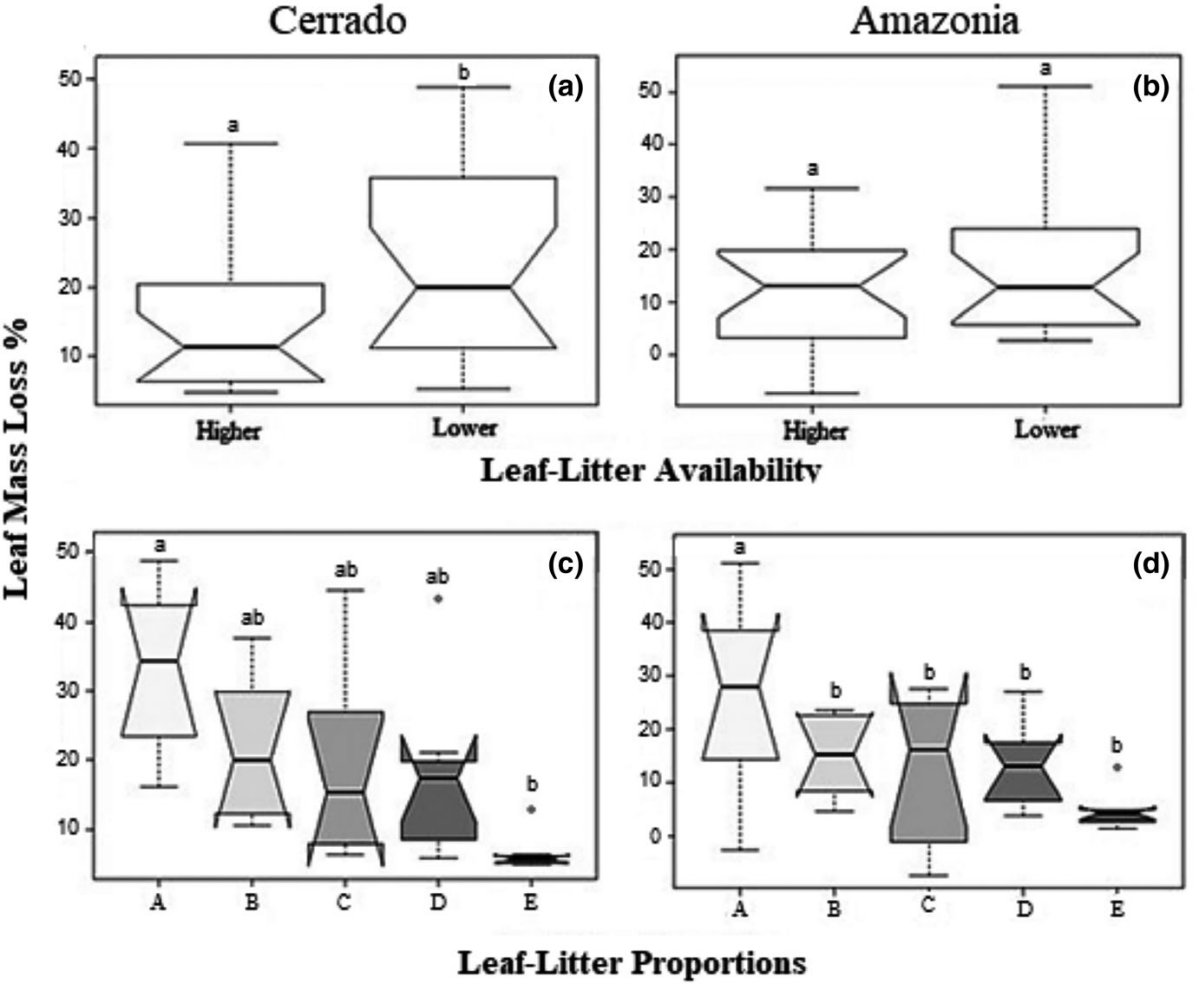
Leaf litter consumption by *Phylloicus* spp. from Cerrado (a) and (c) and from Amazonia (b) and (d), performing the Quantity test (a) and (b) and the Quality test (c) and (d). Treatments: a = 100% of *Maprunia guianensis*; b = 75% of *M. guianensis* and 25% of *Inga laurina*; c = 50% of both; d = 25% of *M. guianensis* and 75% of *I. laurina*; and e = 100% of *M. guianensis.* Boxes represent the quartiles, the bold line represents the mean, and the horizontal dashed line represents the upper and lower limits of variation and circles the outliers

The consumption by *Phylloicus* between Cerrado and Amazonia was statistically different (*F*_1,60_ = 4.95; *p* = .029), as well leaf litter proportions (*F*_4,60_ = 10.85 *p* < .001), and resource availability (*F*_1,60_ = 8.15; *p* = .005). We did not record a significant interaction effect between biomes, leaf litter proportions and the resource availability on *Phylloicus* spp. consumption (Table 1). In Cerrado, higher consumption was recorded in treatment with lower leaf litter quantity (24.0 ± 2.2% standard error) compared with higher quantity (15.0% ±1.7; Figure 3). We recorded higher consumption by *Phylloicus* sp. larvae in treatments with 100% of *M. guianensis* (A; 33.0 ± 3.7%). Treatments with 75% of *M. guianensis* (B—22.0 ± 3.2%), 50% of both, (C—19.0 ± 4.2%), and 25% of *M. guianensis* (D—18.0 ± 3.7%) did not differ in the mass loss. The lower consumption was recorded in 100% of *I. laurina* (E; 7.0 ± 0.8%; Figure 3).In Amazonia, we recorded a consumption of 16.9 ± 4.3% in treatment with lower quantity; and 11.7 ± 3.5% in higher quantity treatment. Similar to Cerrado experiment, we recorded higher consumption in the treatment with 100% of *M. guianensis* (A—27.0 ± 5.5%). However, other treatments did not differ statistically among themselves (B—15.0 ± 5.5%; C—12.0 ± 2.3%; D—13.0 ± 2.4%, and E—5.0 ± 1.1%).

**Table 1:** Summary results table of comparisons between sites, resource availability (leaf quantity), litter proportions (leaf quality) and interaction of this factors (as a linear regression model); and the contrast analysis (*p* < .05) for food resource use by *Phylloicus*, performed at Cerrado and Amazonian treatments

	*df*	Sum. Sq.	*F* value	*p*
Resource availability	1,60	0.089	8.159	**.005**
Leaf litter proportions	4,60	0.476	10.854	**<.001**
Biomes	1,60	0.054	4.956	**.029**
Availability × proportions	4,60	0.035	0.807	.525
Availability × biomes	1,60	0.004	0.385	.537
Proportions × biomes	4,60	0.007	0.166	.954

Note

Bold = significant value.

Abbreviations: *df*, Degrees of freedom; Sum. Sq., Sum of squares.

## 4 DISCUSSION

In general, our results indicated that leaf litter quality were the main factor to explain consumption by *Phylloicus* larvae, supporting our first hypothesis. In both systems, we recorded higher consumption in treatments with only *M. guianensis* leaf litter. Leaf litter consumption of *M. guianensis* in Cerrado (33%), and Amazonia (27%) systems was similar to previous study with this plant species in a Cerrado stream (37%; Rezende et al., 2018). Lignin:N is a worldwide proxy for leaf litter decomposability (Zhang et al., 2019), thus lower Lignin:N and consequently lower hardness explains an accelerated fragmentation by *Phylloicus* (Biasi et al., 2019). Our results corroborate the knowledge that leaf traits are important for the processing of organic matter (Gonçalves et al., 2017; Rezende et al., 2014, 2018). Therefore, changes in plant species composition of riparian zones and, consequently, leaf litter traits may lead to modifications in litter decay, affecting the functioning of Cerrado and Amazonian streams.

The consumption of *I. laurina* leaf litter treatment differed between others only in Cerrado, but not in Amazonian system. These differences in consumption of *I. laurina* were likely due to the greater feeding selectivity of *Phylloicus* on higher quality litter in Cerrado streams than in Amazonian streams. Recalcitrant compounds (e.g., lignin and cellulose) have a negative influence on colonization of decomposing organisms in Cerrado systems (Casotti, Kiffer, & Moretti, 2014; Rezende et al., 2018). Lower quality litter in Cerrado hinders microbial colonization (Sales et al., 2015), and as consequence affected invertebrate consumption (Graça et al., 2015). Furthermore, the low contribution of invertebrate decomposers in tropical streams to leaf litter breakdown is often related to low leaf quality (Rezende et al., 2015). Added the low palatability of this litter species, the low availability of dissolved nutrients limits the litter decomposition in certain Cerrado streams (Alvim, Medeiros, Rezende, & Gonçalves, 2015; Medeiros, Callisto, Graça, Ferreira, & Gonçalves, 2015). This implies directly in the preference of foliar consumption of *Phylloicus* spp. by leaf litter with less structural and secondary compounds and higher nutritional content (Moretti et al., 2009; Rezende et al., 2018; Rincón & Martínez, 2006).

On the other hand, our results showed that different mixing proportions between the two leaf litter species had the same consumption response, but unlike when offered separately. These intermediate leaf proportions had the effect of composition of leaf litter mixtures, which may determine decomposition (Rezende et al., 2016, 2017). Different leaf species have different chemical characteristics (Graça et al., 2015); such interspecific differences have substantial effects on leaf breakdown rates (López‐Rojo et al., 2018). In a mixture of labile and refractory litter species, shredders focused more on the most labile litter species (Swan & Palmer, 2006). However, due to effect of refractory compounds of harder leaf species, equalizing the decay of the entire mixture (López‐Rojo et al., 2018; Navarro & Gonçalves, 2017).

The higher litter availability decreased the use of *I. laurina* leaf litter in microcosm of the Cerrado Amazonia regions, because *Phylloicus* spp. larvae selected softer leaf litter proportions in detriment to less palatable litter for feeding. Although *I. laurina* has higher nutritional content than *M. guianensis*, the processing of softer leaf litter appears to be more cost benefit for *Phylloicus* larvae metabolism (Arias‐Real, Menéndez, Abril, Oliva, & Muñoz, 2018), corroborating our second hypothesis. We recorded a higher consumption in treatment with lower quantity compared with higher quantity just in Cerrado system. The higher quantity of resources may cause a smaller stimulus of resources use to satisfy shredders requirements, according to the concept of order of resource selection (Johnson, 1980). Apparently, *Phylloicus* from Cerrado presents higher stimulus for leaf consumption when there is a smaller litter quantity. Mainly because a vital component to the consumer may be so abundant that the consumer needs only small amounts of it to satisfy their requirement (Johnson, 1980). During periods of less organic matter input in Cerrado streams (Tonin et al., 2017), *Phyllocus* may have this behavior. The lower consumption of both leaf litter in the Amazonian system may be related to the low quality of them for *Phylloicus* from Amazonia (Martins, Melo, et al., 2017) highlighting the importance of leaf traits (e.g., C, N and lignin) for the consumption by shredders (Moretti et al., 2009; Rincón & Martínez, 2006) and leaf litter breakdown process (García‐Palacios, McKie, Handa, Frainer, & Hättenschwiler, 2016). This found reinforces the indication of leaf‐ traits as the considerable factor for leaf breakdown in Amazonian streams (Gonçalves et al., 2017), while the availability of leaf litter does not.

For most containers, mainly *M. guianensis* was used (fragment) in case building. *Phylloicus* larvae prefer leaves with low quality for case building (Moretti et al., 2009; Rincón & Martínez, 2006), then, *M. guianensis* is not ideal for case building, because it has low concentration of refractory compounds compared with *I. laurina*. However, its fragmentation is easier than litter with lower quality and greater hardness (Gonçalves et al., 2017). In treatment where there was only litter of *I. laurina*, we noted the use of sand from the bottom of the container for case building. This fact could be a response for *I. laurina* toughness*,* because larvae have more energy cost to fragment *I. laurina* affecting the fragmentation process of leaf litter (Moretti et al., 2009; Biasi et al., 2019). *Inga laurina* traits may have induced *Phylloicus* to use the fine substrate from the bottom of the containers for case construction, instead of using its leaf. Such behavior may have negative effects on the decomposition of organic matter, because reduces its processing.

## 5 CONCLUSION

As many others experiments in laboratory, our study synthesizes the natural environment in search of answers that can add to the knowledge about the role of the organic matter breakdown process. Although *Phylloicus* larvae from Amazon and Cerrado are possibly distinct species, our results indicated that leaf traits (as lignin:N content) drives the preference of litter consumption by *Phylloicus* larvae from both biomes streams. Moreover, we showed how the availability of leaf litter might affect its use by *Phylloicus* in the Cerrado, in opposition to the Amazonian. In this way, it is possible to conclude that different resource availability, tends to be more marked for *Phylloicus* from Cerrado streams. Finally, we suggest that a possible change in plant composition might have a direct effect on the processing of organic matter in tropical streams.

## AUTHOR CONTRIBUTIONS

**Guilherme Sena**: Writing‐original draft (lead); writing‐review and editing (equal). **José Francisco Gonçalves Júnior**: Supervision (equal); writing‐review and editing (equal). **Renato Tavares Martins**: Conceptualization (equal); data curation (equal); methodology (equal); writing‐review and editing (equal). **Neusa Hamada**: Supervision (equal); writing‐review and editing (equal). **Renan de Souza Rezende**: Conceptualization (equal); data curation (equal); formal analysis (equal); methodology (equal); writing‐review and editing (equal).

## Data Availability

The data that support the findings of this study is submitted in the Dryad public repository: https://doi.org/10.5061/dryad.4j0zpc871
